# Efficacy of Bracket Adhesive Remnant Removal by a Fluorescence-Aided Identification Technique with a UV Light Handpiece: *In Vitro* Study

**DOI:** 10.1155/2022/4821021

**Published:** 2022-09-19

**Authors:** Sabrina Elise Moecke, Pedro Caio Alves Barros, Adrielle Caroline Moreira Andrade, Alessandra Bühler Borges, César Rogério Pucci, Carlos Rocha Gomes Torres

**Affiliations:** Department of Restorative Dentistry, Institute of Science and Technology, Sao Paulo State University-UNESP, Sao Jose dos Campos, SP, Brazil

## Abstract

**Objective:**

This study aims to analyze the fluorescence-aided identification technique efficacy on adhesive remnant removal from the enamel surface after orthodontic bracket debonding.

**Materials and Methods:**

Forty-five extracted human upper central incisors were divided into 3 groups (*n* = 15) according to the kind of adhesive for bracket bonding and the use or absence of near UV light for remnant removal: BF/UV- fluorescent adhesive/UV light, BF/0-fluorescent adhesive/no UV light, and TB/0-nonfluorescent adhesive/no UV light. For all teeth, 100% of the adhesive used remained on the enamel surface after debonding. Fifteen dentists performed adhesive removal on the enamel surface using a carbide bur. The specimens were analyzed by a stereomicroscope, and the adhesive remnant percentage from each specimen was calculated. The time used by each dentist to perform the removal was recorded. The data were analyzed by one-way ANOVA and Tukey's test.

**Results:**

Significant differences were observed among groups for adhesive remnant (*p*=0.0008) and for time (*p*=0.0001). The means of adhesive remnant were BF/UV (5.84), BF/0 (34.37), and TB/0 (37.02). The mean times necessary to remove adhesive were BF/UV (1 min 40 s), BF/0 (3 min 03 s), and TB/0 (2 min 46 s). For the BF/UV group, significantly lower values of adhesive remnants and time for debonding were found (*p* < 0.05).

**Conclusion:**

The fluorescence-aided identification technique significantly reduced the amount of adhesive remnant, and the time necessary to perform this clinical procedure.

## 1. Introduction

Traditionally, orthodontic treatment includes the use of metallic or esthetic brackets, which are bonded to enamel generally using a resin-based adhesive [1]. After achieving the desired tooth repositioning, the brackets need to be safely removed, as well as all residues of adhesive that remain on the surface [2]. Since the bonding material has a color similar to the tooth, it can be a challenge to differentiate it from the enamel with the naked eye and perform complete removal without damaging the enamel [2, 3].

Before the debonding procedure, it is necessary to understand how the adhesion protocol can influence the bond results. The bracket stability during the orthodontic treatment is the visible achievement of the ideal bond strength. It is possible that in addition to the total or self-etching procedure, a clean tooth surface can improve bond strength [4]. To improve the bond strength, several studies have tested different techniques, including enamel pretreatment, such as air abrasion [5, 6] and abrasive pastes [4]. The etching procedure as total or self-etching can modify how the etch pattern and the resin tag infiltration occur [7, 8], which can be relevant for later debonding and adhesive remnant removal procedures.

Several studies were performed with the intention of determining an effective method for adhesive remnant removal after bracket debonding [1, 2, 9–12]. The results showed that no technique or instrument until now was able to completely remove the adhesive without causing damage [1, 11]. Therefore, ways of improving this procedure are relevant for achieving safer orthodontic treatment. Ideally, adhesive remnant removal should not harm the enamel, leave the minimum or no residues on the surface, and be easily and quickly performed. Time is a relevant aspect, since there are usually several teeth needing the procedure [13].

The fluorescence-aided identification technique (FIT) has been developed to facilitate the visualization of differences in fluorescence levels between the tooth structure and the dental material [14–17]. Recently, the method was adapted for the recognition of orthodontic adhesives [18–22]. Fluorescence is defined as the property of absorbing short wavelength light and emitting longer wavelength light, which can or cannot be seen [23]. This phenomenon occurs in natural teeth [24, 25], and for sound enamel, the range of light emission is 430–450 nm, which is usually in a different color or intensity from dental material, making it possible to distinguish the fluorescence emission of the tooth structure from the material [16, 26]. Therefore, when fluorescent emission is used to detect adhesive remnants, it is desired that the bracket adhesive shows a fluorescent level higher than the intact tooth [13, 18–22, 27–29]. In this way, some manufacturers developed special bracket adhesive materials with high fluorescence levels to facilitate their detection using FIT.

Generally, the dentist uses a separate ultraviolet (UV) light source to illuminate the tooth where the bracket remnants are intended to be removed [18, 21, 22]. However, some manufacturers developed high-speed handpieces with integrated light outlets near the head [30], including UV light, improving the access of the light to the active tip of the bur during the procedure [19, 20]. UV handpieces seem to be the best option for the removal of new highly fluorescent adhesive materials for brackets. However, there are a limited number of studies available to provide solid scientific evidence of the efficacy and advantages of this combination.

This study aimed to analyze the effects of FIT on adhesive remnant removal efficacy and time required using a dedicated fluorescent orthodontic adhesive and a UV handpiece. The null hypothesis was that the FIT would not affect the remnant removal and the time to perform the procedure.

## 2. Materials and Methods

The Research Ethics Committee approved the protocol of this study (CAAE N 15289019.1.0000.0077). The sample size was calculated assuming adhesive remnant percentage as the primary outcome with an *a* = 0.05 and power = 80%. According to a pilot study, performed using the same method applied in the actual research, the expected mean difference was 28, 2; therefore, 15 teeth per group were needed. Fifteen dentists (8 female and 7 male) were also enrolled in the study. Each participant was informed of its objectives and provided informed consent.

### 2.1. Specimen Preparation

Forty five intact human upper central incisors, extracted for periodontal reasons, were stored in 0.1% thymol solution at 5°C until needed. Teeth surfaces were cleaned by scalpel blade and ultrasonic scaler as needed and with pumice on a rotary brush and stored in ultrapure water before use. To allow all bracket adhesive applied to remain over the tooth structure after debonding, the internal base physical retention of the metallic brackets (Edgewise Standard. 022″11, 21, Morelli, Sorocaba, SP, Brazil) was closed with melted wax [10].

Two different light-cured bracket bonding systems were tested. Thirty teeth received the fluorescence under a UV light (BrackFix, Voco, Cuxhaven, Germany), while fifteen received regular material without any fluorescence (Transbond XT, 3M/ESPE, San Paul, USA). For BrackFix, a self-etching primer was actively applied over the enamel surface for 5 s [31]. For Transbond XT, a previous etching with a 35% phosphoric acid gel was performed for 15 s [32]. After that, the surface was washed and air dried, and the primer was applied. The respective adhesives were applied on the bracket base, which was placed in position on the center of the labial surface of the crowns. The material excess at the bracket edges was carefully removed [32]. Light-curing was performed with an LED device (Valo, Ultradent, South Jordan, UT, USA) for 10 s mesially and 10 s distally [31]. The specimens were all prepared by a single operator, and then stored in ultrapure water for 24 h. The debonding was performed by a single operator using an orthodontic plier.

**Figure 1:**
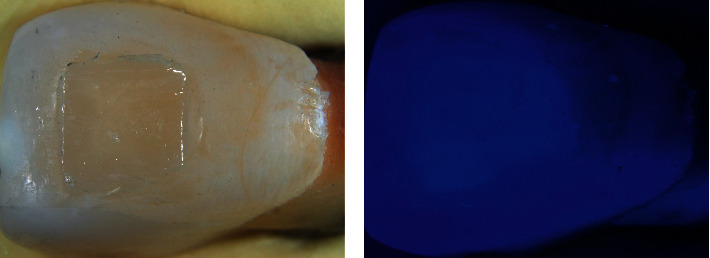
Adhesive remnant area analysis. (a) Adhesive after debonding; (b) fluorescent adhesive remnant under UV light.

All specimens were analyzed by a stereomicroscope (Discovery V20, Carl Zeiss, Jena, Germany) with a 1.0× lens and 9.0× magnification (Figure 1(a)). The area occupied by the adhesive remnants was measured (mm^2^) using the image-processing and analysis software Zen 2 Blue Edition (Carl Zeiss, Jena, Germany).

### 2.2. Adhesive Removal

To simulate a real clinical condition during the adhesive removal procedure, a dental training model (MOM, Marilia, SP, Brazil) placed inside a dental simulator phantom (MOM) was used. The natural teeth containing the adhesive remnants were placed in the central incisor position, one at a time. The specimens were identified only on the root surface, which was not visible to operators.

The dental chair light was turned on, and the dentists enrolled in the study were requested to remove the adhesive remnants on the surface using a 30-fluted bullet shape carbide bur (9803FF, KG Sorensen, Sao Paulo, SP, Brazil) with air/water spray [1, 11]. The mean age of the dentists who performed the procedures was 35.07 ± 10.44, and the mean years of experience was 10.33 ± 10.55. The bur was attached to a high-speed handpiece (Cobra LED Ultra Vision, Gnatus, Ribeirao Preto, SP, Brazil) coupled with a near UV light source (405 nm wavelength, 25.000 lux) [20]. During use, near UV light could be turned on or off as the group that was being performed. A new bur was used for each tooth. An exploratory probe was also available for inspection of remnants according to the dentist's wishes. The decision about when the remnants were completely removed was based on the personal opinion of each clinician. The time necessary to remove the remnants on each tooth was recorded using a chronometer, starting from the moment the dentist had the first look to the tooth surface. Then, the tooth was removed and stored in water until the analysis was performed. A new one was placed in the same position, and the dentist repeated the procedure, according to the group. Each dentist removed the adhesive of three teeth, one from each group. The order of the groups was previously randomized.

For Transbond XT, the adhesive was removed without a light source for all 15 specimens (TB/0). For BrackFix, the specimens were divided into two subgroups (*n* = 15). In the first one (BF/0), adhesive removal was performed the same way as described, without a light source. For the second one (BF/UV), the dentists were requested to remove the adhesive remnant similar to what was performed before, but this time with the near UV light on.

### 2.3. Analysis of Adhesive Remnant

After the removal procedure, new pictures were obtained by stereomicroscopy, and the area occupied by the remnants was measured. Considering the total area on the baseline as 100%, the percentage of the remnant area was calculated [17]. To clearly identify the adhesive remnants, pictures of the specimens that received the fluorescent adhesive (BF/UV and BF/0 groups) were obtained under a UV light source (Figure 1(b)).

**Figure 2:**
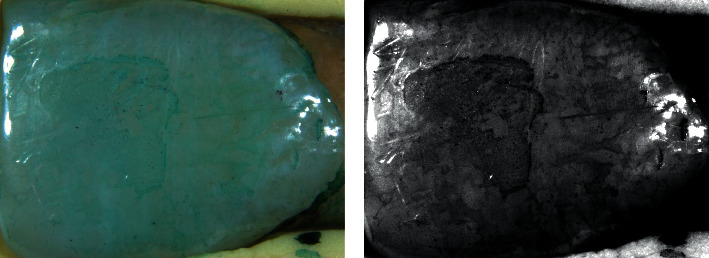
Adhesive remnant area analysis. (a) Green-stained tooth treated with nonfluorescent adhesive under white light; (b) image treated in Photoshop for better adhesive remnant identification.

For the specimens bonded with the nonfluorescent adhesive (TB/0), the surface received the application of a green dye (Sable Seek Green, Ultradent, South Jordan, UT, USA) for 10 s, followed by washing with an air/water spray and drying with an air stream. The pictures were obtained under a white light source (Figure 2(a)). After that, the image was processed using image editing software (Photoshop, Adobe, San Jose, CA, EUA), changing the contrast, saturation, and hue to provide a better remnant visualization (Figure 2(b)).

### 2.4. Statistical Analysis

The normality of the data was checked by the Shapiro-Wilk test. The comparison among the groups was performed using one-way ANOVA and Tukey's test for adhesive remnant percentage and time necessary to perform removal. A significance level of 5% was adopted.

## 3. Results

**Figure 3:**
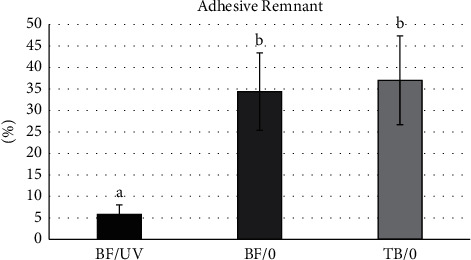
Means (SD) of adhesive remnant percentage for all groups and results of Tukey's test. Different letters in columns show significant differences.

The results of one-way ANOVA showed significant differences for adhesive remnant percentage (*p*=0.00085).  Figure 3 shows that the BF/UV group showed significantly less adhesive remnant than the others. No significant differences were found between the BF/0 and TB/0 groups.

Significant differences for the time used in adhesive removal were shown by one-way ANOVA (*p*=0.0013). The group in which the near UV light was used required significantly less time for adhesive removal. The groups without light source did not show significant differences.

## 4. Discussion

The adhesive remnant area after removal and the time necessary to perform the procedure were significantly reduced when fluorescent adhesive was combined with near UV light, allowing rejection of the null hypothesis. This is in agreement with previous studies [18, 20, 22, 27], which demonstrated that a better adhesive remnant visualization may improve its removal. The permanence of adhesive residue may have a negative impact on caries prevention and long-term esthetic outcomes [27]. The remnants are salient areas that can contribute to biofilm accumulation [10] and staining [33, 34].

Ideal adhesive removal should leave nothing or as little adhesive as possible on the surface, in combination with no enamel damage. Although it is clear that fixed orthodontic treatment causes some level of irreversible damage to dental enamel [1, 19], FIT might make remnant removal safer [16, 18, 22]. The carbide bur used in the present study was used to perform adhesive removal in several previous studies [2, 10, 19–21, 27, 34, 35], since it provides effective and safer removal in comparison with other rotary instruments, such as diamond points, with an acceptable clinical time [36]. In addition to the instrument used, the enamel damage magnitude is operator dependent [2, 18], and it has been observed even when FIT was used [21]. To reduce operator interference, fifteen dentists were recruited in the present study to perform the removal procedure. Each one decided based on their own experience, under examination using an exploratory probe [2], when they considered the residue well removed. Since the aim of this study was the effect of FIT, the polishing step was not included, even though it is well established that remnant removal should be followed by polishing techniques for a smoother surface [1, 9, 19].

The time necessary to remove the remnant is a controversial step, since some techniques and instruments can perform well in a certain aspect, but they take so much time to result in acceptable removal that they are not used by dentists on a regular basis [37]. A way to provide better visualization, such as FIT, can reduce the clinical time and provide better cost-effectiveness, which eventually leads to higher dentist acceptance [19]. For some patients, time is even more important, as for children and elderly individuals, or any other person who has difficulties or pain when keeping their mouth open for longer periods [19].

**Figure 4:**
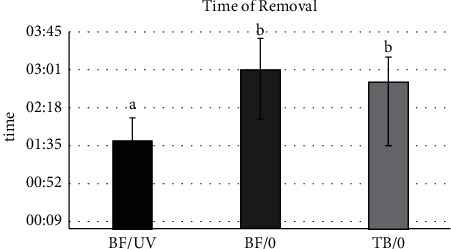
Means (SD) of the time used by the dentists to remove the adhesive remnant for all groups and results of Tukey's test. Different letters in columns show significant differences.

This study showed that a near UV light source in combination with a fluorescent adhesive significantly reduced the time necessary to remove adhesive when compared to the removal without light (Figure 4). The time was increased by more than a minute when the removal was done without UV. This difference is even more relevant when considering the necessity to repeat the procedure in several teeth. Therefore, the previously recommended instrument for safer and faster removal, the 30-fluted carbide bur [1, 36], in combination with a near UV light, may further improve the time effectiveness of this procedure.

Enamel loss was previously reported to be increased by FIT [29], which might be attributed to the visualization of fluorescence from the resin tags on enamel. The resin tags of total etching protocols were reported to have up to 50 *μ*m depth [7, 8], while the self-etching primers have demonstrated a shallower etch pattern and resin tag infiltration [8, 38]. In contrast to a previous study [29], in the present study, the fluorescent adhesive was a self-etching primer, which may contribute to a more conservative etch pattern than when performed with phosphoric acid, minimizing enamel loss [38].

A previous analysis suggested that a fluorescent adhesive with a thickness smaller than 2 *μ*m was not detectable under UV illumination [18], and another study demonstrated that from 800 to 50 *μ*m thickness, the fluorescence distinction between adhesive and adjacent teeth was improved by UV light [28]. However, since the fluorescent emission of the adhesive tested in this study was never measured before, the results obtained with other materials cannot be transferred to the one tested here. FIT has a high sensitivity and specificity [20], and thus, UV light and fluorescence contribute to better and faster adhesive removal and less enamel damage [3, 18, 20, 21, 27]. Despite the favorable results, some limitations can be noted, such as the use of a single method to perform the removal, the lack of enamel damage analysis after removal, and ultrastructural analysis by scanning electron microscopy. The use of different operators to perform the removal was a strategy for observing distinct operators' results, although it can also be a limitation of this study. Moreover, the results of this in vitro study need confirmation from future clinical trials.

## 5. Conclusions

It can be concluded that the amount of adhesive remnant was significantly smaller when the fluorescent adhesive BrackFix was used, and the removal was performed with a handpiece associated with a near UV light source performing the fluorescent-aided identification technique. This combination also resulted in time reduction to perform the procedure.

## Data Availability

The data used to support the findings of this study are available from the corresponding author upon request.

## Additional Points

All applicable patient releases were obtained and are on file with the corresponding author.

## References

[1] Janiszewska-Olszowska J., Szatkiewicz T., Tomkowski R., Tandecka K., Grocholewicz K. (2014). Effect of orthodontic debonding and adhesive removal on the enamel-current knowledge and future perspectives-a systematic review. *Medical Science Monitor*.

[2] Bonetti G. A., Zanarini M., Parenti S. I., Lattuca M., Marchionni S., Gatto M. R. (2011). Evaluation of enamel surfaces after bracket debonding: an *in-vivo* study with scanning electron microscopy. *American Journal of Orthodontics and Dentofacial Orthopedics*.

[3] Rossato P. H., Kaneshima E. N., Domingues F., Fernandes T. M. F., Berger S. B., Oltramari P. V. P. (2020). Do fluorescent agents alter the mechanical strength of orthodontic adhesives? An *in vitro* and clinical study. *Progress in Orthodontics*.

[4] Talic N. F. (2011). Effect of fluoridated paste on the failure rate of precoated brackets bonded with self-etching primer: a prospective split-mouth study. *American Journal of Orthodontics and Dentofacial Orthopedics*.

[5] Scribante A., Gallo S., Pascadopoli M., Catalano F., Gandini P., Sfondrini M. F. (2022). Effect of different enamel pretreating agents on bonding efficacy and survival rates of orthodontic brackets: in vitro study and split-mouth randomized clinical trial. *American Journal of Orthodontics and Dentofacial Orthopedics*.

[6] Huilcapi M., Armas-Vega A., Cardenas A. F. M. (2020). Effect of surface treatments on the adhesive properties of metallic brackets on fluorotic enamel. *Dental Press Journal of Orthodontics*.

[7] Silverstone L., Saxton C. A., Dogon I. L., Fejerskov O. (1975). Variation in the pattern of acid etching of human dental enamel examined by scanning electron microscopy. *Caries Research*.

[8] Ramesh Kumar K. R., Shanta Sundari K. K., Venkatesan A., Chandrasekar S. (2011). Depth of resin penetration into enamel with 3 types of enamel conditioning methods: a confocal microscopic study. *American Journal of Orthodontics and Dentofacial Orthopedics*.

[9] Ulusoy Ç. (2009). Comparison of finishing and polishing systems for residual resin removal after debonding. *Journal of Applied Oral Science*.

[10] Ahrari F., Akbari M., Akbari J., Dabiri G. (2013). Enamel surface roughness after debonding of orthodontic brackets and various clean-up techniques. *Journal of Dentistry*.

[11] Lima Ferreira J. T., Pereira Saraiva M. C., Nakane Matsumoto M. A., Romano F. L., Borsatto M. C., Torres C. P. (2020). Evaluation of enamel roughness *in vitro* after orthodontic bracket debonding using different methods of residual adhesive removal. *Turkish Journal of Orthodontics*.

[12] Smith S. C., Walsh L. J., Taverne A. A. R. (1999). Removal of orthodontic bonding resin residues by CO_2_ laser radiation: surface effects. *Journal of Clinical Laser Medicine and Surgery*.

[13] Namura Y., Tsuruoka T., Ryu C., Kaketani M., Shimizu N. (2010). Usefulness of orthodontic adhesive-containing fluorescent dye. *The European Journal of Orthodontics*.

[14] Kiran R., Chapman J., Tennant M., Forrest A., Walsh L. J. (2019). Detection of tooth-colored restorative materials for forensic purposes based on their optical properties: an *in vitro* comparative study. *Journal of Forensic Sciences*.

[15] Kiran R., Walsh L. J., Forrest A., Tennant M., Chapman J. (2017). Forensic applications: fluorescence properties of tooth-coloured restorative materials using a fluorescence DSLR camera. *Forensic Science International*.

[16] Meller C., Connert T., Löst C., ElAyouti A. (2017). Reliability of a fluorescence-aided identification technique (FIT) for detecting tooth-colored restorations: an ex vivo comparative study. *Clinical Oral Investigations*.

[17] Dettwiler C., Eggmann F., Matthisson L., Meller C., Weiger R., Connert T. (2020). Fluorescence-aided composite removal in directly restored permanent posterior teeth. *Operative Dentistry*.

[18] Lai C., Bush P. J. B., Warunek S., Covell D. A., Al-Jewair T. (2019). An *in vitro* comparison of ultraviolet versus white light in the detection of adhesive remnants during orthodontic debonding. *The Angle Orthodontist*.

[19] Kaneshima E. N., Berger S. B., Fernandes T. M. F., Navarro M. F. L., Oltramari P. V. P. (2018). Using UV light for adhesive remnant removal after debonding of orthodontic accessories. *Brazilian Oral Research*.

[20] Schott T. C., Meller C. (2018). A new fluorescence-aided identification technique (FIT) for optimal removal of resin-based bracket bonding remnants after orthodontic debracketing. *Quintessence International*.

[21] Stadler O., Dettwiler C., Meller C., Dalstra M., Verna C., Connert T. (2019). Evaluation of a Fluorescence-aided Identification Technique (FIT) to assist clean-up after orthodontic bracket debonding. *The Angle Orthodontist*.

[22] Albertini P., Albertini E., Siciliani G., Lombardo L. (2020). Fluorescence-aided composite removal during lingual bracket debonding. *Journal of Esthetic and Restorative Dentistry*.

[23] Lakowicz J. R. (2009). *Principles of Fluorescence Spectroscopy*.

[24] Da Silva R. D., da Silva M. A. D., de Oliveira O. B., Melo A. C. M., de Oliveira R. N. (2013). Dental fluorescence: potential forensic use. *Forensic Science International*.

[25] Baratieri L. N., Araujo E., Monteiro S. (2007). Color in natural teeth and direct resin composite restorations: essential aspects. *The European Journal of Esthetic Dentistry*.

[26] Takahashi M. K., Vieira S., Rached R. N., Almeida J. B., Aguiar M., Souza E. M. (2008). Fluorescence intensity of resin composites and dental tissues before and after accelerated aging: a comparative study. *Operative Dentistry*.

[27] Ribeiro A. A., Almeida L. F., Martins L. P., Martins R. P. (2017). Assessing adhesive remnant removal and enamel damage with ultraviolet light: an *in-vitro* study. *American Journal of Orthodontics and Dentofacial Orthopedics*.

[28] Kim G. M., Kim B. R., Lee E. S., de Josselin de Jong E., Kim B. I. (2018). Detection of residual resin-based orthodontic adhesive based on light-induced fluorescence. *Photodiagnosis and Photodynamic Therapy*.

[29] Rocha R. S., Salomão F. M., Silveira Machado L., Sundfeld R. H., Fagundes T. C. (2017). Efficacy of auxiliary devices for removal of fluorescent residue after bracket debonding. *The Angle Orthodontist*.

[30] Walsh L. J., Mandikos M. N. (2015). *Illumination Dental Instrument, Coupling and Method of Use - United States Patent US9028257B2*.

[31] Knaup I., Weber E., Böddeker A. (2021). Effect of using different component combinations for orthodontic bracket bonding with self-etch primers. *Journal of Orofacial Orthopedics*.

[32] Özer T., Başaran G., Kama J. D. (2010). Surface roughness of the restored enamel after orthodontic treatment. *American Journal of Orthodontics and Dentofacial Orthopedics*.

[33] Sundfeld R. H., Franco L. M., MacHado L. S. (2016). Treatment of enamel surfaces after bracket debonding: case reports and long-term follow-ups. *Operative Dentistry*.

[34] Joo H. J., Lee Y. K., Lee D. Y., Kim Y. J., Lim Y. K. (2011). Influence of orthodontic adhesives and clean-up procedures on the stain susceptibility of enamel after debonding. *The Angle Orthodontist*.

[35] Sadrhaghighi A. H., Mohammadi A., Abdollahzadeh Baghaei T., Alipour H. (2020). Tooth color alteration after debonding in orthodontic patients with adhesive removal using composite bur or tungsten carbide bur: a single center, randomized controlled clinical trial. *Brazilian Dental Science*.

[36] Zarrinnia K., Eid N. M., Kehoe M. J. (1995). The effect of different debonding techniques on the enamel surface: an in vitro qualitative study. *American Journal of Orthodontics and Dentofacial Orthopedics*.

[37] Karan S., Kircelli B. H., Tasdelen B. (2010). Enamel surface roughness after debonding Comparison of two different burs. *The Angle Orthodontist*.

[38] Cal-Neto J. P., Miguel J. A. M. (2006). Scanning electron microscopy evaluation of the bonding mechanism of a self-etching primer on enamel. *The Angle Orthodontist*.

